# The Administration of 4-Hexylresorcinol Accelerates Orthodontic Tooth Movement and Increases the Expression Level of Bone Turnover Markers in Ovariectomized Rats

**DOI:** 10.3390/ijms21041526

**Published:** 2020-02-24

**Authors:** Kwang-Hyo Choi, Dae-Won Kim, Suk Keun Lee, Seong-Gon Kim, Tae-Woo Kim

**Affiliations:** 1 Department of Orthodontics, College of Dentistry, Seoul National University, Seoul 3080, Korea; joshuakh@hotmail.com; 2 Department of Oral Biochemistry, College of Dentistry, Gangneung-Wonju National University, Gangneung 28644, Korea; kimdw@gwnu.ac.kr; 3 Department of Oral Pathology, College of Dentistry, Gangneung-Wonju National University, and Institute of Oral Science, Gangneung 28644, Korea; 4 Department of Oral and Maxillofacial Surgery, College of Dentistry, Gangneung-Wonju National University, Gangneung 28644, Korea

**Keywords:** animal model, bone morphogenic protein, 4-hexylresorcinol, nuclear factor kappa-B ligand, orthodontic, ovariectomy, tooth movement

## Abstract

Surgical methods for accelerating orthodontic tooth movement are limited by possible damage to the tooth root and patient discomfort. 4-Hexylresorcinol (4HR) has been shown to increase bone remodeling and may potentially facilitate tooth movement. This study investigated the (1) effect of 4HR administration on osteoblast-like cells and (2) effect of 4HR administration on tooth movement in ovariectomized rats. Saos-2 cells were treated with either 4HR or solvent (control). Protein expression levels were investigated 2, 8, and 24 h after treatment. Thirty ovariectomized Sprague-Dawley rats were divided into two experimental groups (A and B) and one control group. After installation of an orthodontic tooth movement device, groups A and B received subcutaneous weekly injections of 4HR (1.28 and 128 mg/kg). Micro-computerized tomography and histological analyses were performed after 2 weeks of tooth movement. The application of 4HR elevated expression of osteogenic markers in Saos-2 cells. Movement of the first molars was significantly greater in rats administered 4HR. Furthermore, the expression of bone morphogenic protein-2, receptor activator of nuclear factor kappa-B ligand, osteocalcin, and tartrate-resistant acid phosphatase were increased after 4HR administration. 4HR application demonstrated increased expression of osteogenic markers in Saos-2 cells and accelerated orthodontic tooth movement in rats.

## 1. Introduction

Orthodontic treatment involves tooth movement, which requires the remodeling of the surrounding tissues. A number of studies have reported the physiological changes that occur in the alveolar bone during the application of orthodontic forces [1,2,3]. In order for tooth movement to occur without damaging the surrounding tissues, the alveolar bone is resorbed on the side towards which the tooth is being moved, and new bone formation occurs on the opposite side [2,3]. While tipping the balance towards bone resorption may accelerate tooth movement, this may also result in periodontal lesions.

Several trials have been conducted to investigate optimal methods to accelerate tooth movement. For example, studies have evaluated the effectiveness of increasing the amount of orthodontic force used [3,4], as well as the use of vibration [5]. Surgical interventions such as corticotomy, micro-perforation, and piezocision have been assessed for their ability to induce accelerated bone remodeling [6,7]. Nevertheless, such procedures may damage the tooth root and cause discomfort and pain. The effects of hormones and drugs on tooth movement have also been investigated [8,9]. Parathyroid hormone has been reported to accelerate orthodontic tooth movement by increasing osteoclastic activity [8]. However, the application of hormones may also disrupt the endocrine system and cause unexpected health problems [10]. Furthermore, the stimulation of bone resorption may result in delayed bone formation on the tension side, leading to periodontal problems.

4-Hexylresorcinol (4HR) is a kind of resorcinolic lipid and synthetics [11]. 4HR has been used in cosmetics and antiseptics without serious complications [12]. According to recent study, 4HR has been incorporated into bone grafts for the inhibition of foreign body giant cell formation [13] and the nuclear factor kappa B pathway [14]. Multinucleated giant cell formation is a key feature for the development of osteoclast [15]. During osteoclastogenesis, the nuclear factor kappa B pathway is activated [16]. Accordingly, 4HR administration has been used for increasing bone formation [17]. 4HR increases angiogenesis via a hypoxia-inducible factor (HIF)-independent pathway, but the detailed mechanism remains unclear [18]. We have recently reported that 4HR increases the expression of transforming growth factor-β1 (TGF-β1) [19]. TGF-β1 is also important in bone regeneration and remodeling [20]. Collectively, 4HR administration may accelerate orthodontic tooth movement without impairing bone formation.

To clarify 4HR induced bone formation in the orthodontic tooth movement, slow tooth movement is not a proper model. The ovariectomized animal model has an increased bone turnover rate; therefore, it has been widely used for the study of osteoporosis [21,22]. Bone turnover rate is closely associated with the speed of the orthodontic tooth movement [23]. The speed of bone formation is slower than that of bone resorption during the rapid orthodontic tooth movement [24]. For the evaluation of orthodontic tooth movement, accurate evaluation of root position is important. Recently, three-dimensional prediction method by computed tomography (CT) shows high accuracy of root position [25]. Accordingly, the ability of catch-up bone formation by 4HR will be demonstrated clearly in the rapid tooth movement model with CT evaluation. The ovariectomized animal model is optimal for studies investigating the effectiveness of treatments aimed at accelerating orthodontic tooth movement and accompanying complications [24].

The objective of this study was to demonstrate (1) the effect of 4HR administration on osteoblast-like cells, and (2) the effect of 4HR administration on tooth movement and the expression level of bone remodeling markers in an ovariectomized animal model.

## 2. Results

### 2.1. 4HR Application Increases Osteogenic Markers in Saos-2 Cells

**Figure 1:**
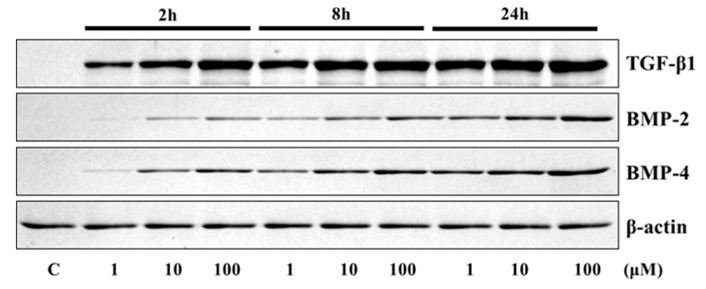
Western blot analysis for transforming growth factor-β1 (TGF-β1), bone morphogenic protein-2 (BMP-2), and BMP-4. Expression levels were increased after 4HR administration.

**Figure 2:**
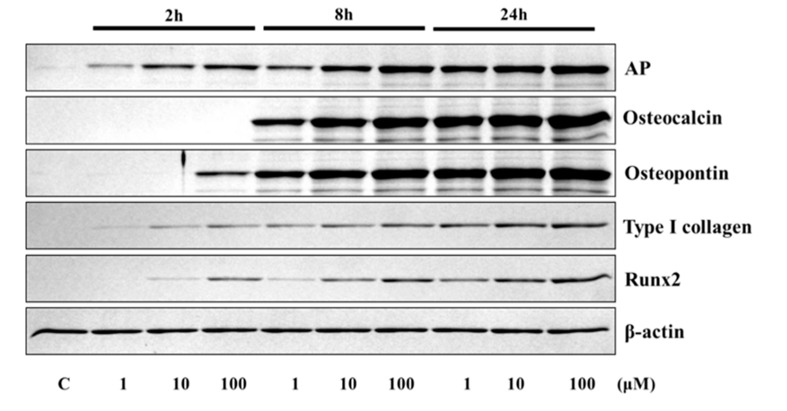
Western blot analysis for alkaline phosphatase (AP), osteocalcin, osteopontin, type I collagen, and runt-related transcription factor 2 (RUNX2). Expression levels were increased after 4HR administration.

The application of 4HR to Saos-2 cells elevated the expression of osteogenic markers (Figure 1 and Figure 2). The expression level of TGF-β1, bone morphogenic protein-2 (BMP-2), BMP-4, alkaline phosphatase (AP), osteocalcin (OC), osteopontin (OP), type I collagen, and runt-related transcription factor 2 (Runx2) were increased after 4HR administration. The increase in expression levels was dose (1–100 μM) and time-dependent (2–24 h). OC and OP were particularly highly increased by 4HR administration.

### 2.2. Effects of 4HR on the Expression of Osteogenesis-Related Proteins in Saos-2 Cells

**Figure 3:**
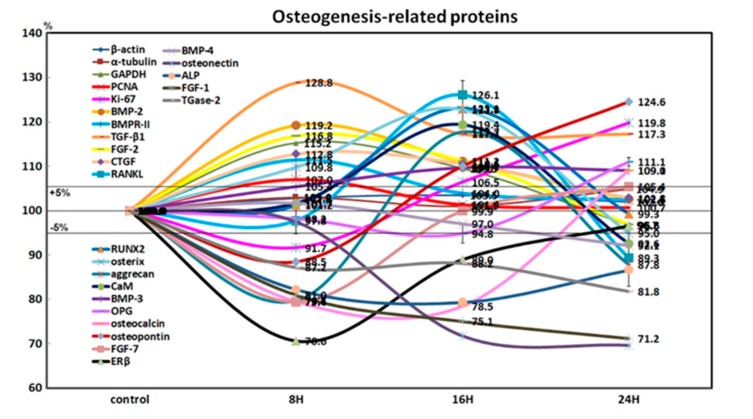
Expression of osteogenesis-related proteins (*n* = 21) in 4HR-treated Saos-2 cells, as determined by high-performance liquid chromatography. The line graph shows protein expression patterns on the same scale (%) versus culture time (8, 16, or 24 h).

4HR-treated Saos-2 cells showed slight increases in the expression of Ki-67 (19.8% at 24 h) and proliferating cell nuclear antigen (7% at 8 h) after 24 h, compared to the non-treated control. This indicated that the cellular proliferation of Saos-2 cells was relatively well-preserved during the 24 h of 4HR treatment (Figure 3).

4HR-treated Saos-2 cells showed sequential dominant expression of osteogenesis-related proteins at 8, 16, and 24 h. Proteins that were overexpressed at 8 h included BMP-2 (19.2%), bone morphogenetic protein receptor-II (BMPR-II, 11.5%), TGF-β1 (28.8%), fibroblast growth factor-2 (FGF-2, 16.8%), and connective tissue growth factor (CTGF, 12.8%), which are relevant to the induction of bone formation. Proteins that were overexpressed at 16 h included RANKL (26.1%), RUNX2 (23.1%), osterix (22.8%), aggrecan (17.7%), and calmodulin (CaM, 19.4%), which are relevant to osteoblast differentiation. Proteins overexpressed at 24 h were BMP-3 (9%), osteoprotegerin (OPG, 11.1%), osteocalcin (9.1%), and osteopontin (24.6%), which are relevant to osteoid matrix deposition (Figure 3).

Furthermore, 4HR-treated Saos-2 cells showed marked downregulation of bone maturation-related proteins, including FGF-7 (20.6%), estrogen receptor β (ERβ, 29.4%), BMP-4 (3%), osteonectin (28.2%), AP (20.6%), FGF-1 (24.9%), and transglutaminase-2 (TGase-2, 12.8%) at 8 and 16 h. At 24 h, there was an upregulation of FGF-7 (5.4%), and slight downregulation of ERβ (3.4%), BMP-4 (7.9%), and AP (13.4%). A continuous marked downregulation of osteonectin (30.3%), FGF-1 (28.8%), and TGase-2 (18.2%) were also observed at 24 h. These data suggested that 4HR-treated Saos-2 cells showed rare bone maturation after 24 h of culture (Figure 3).

**Figure 4:**
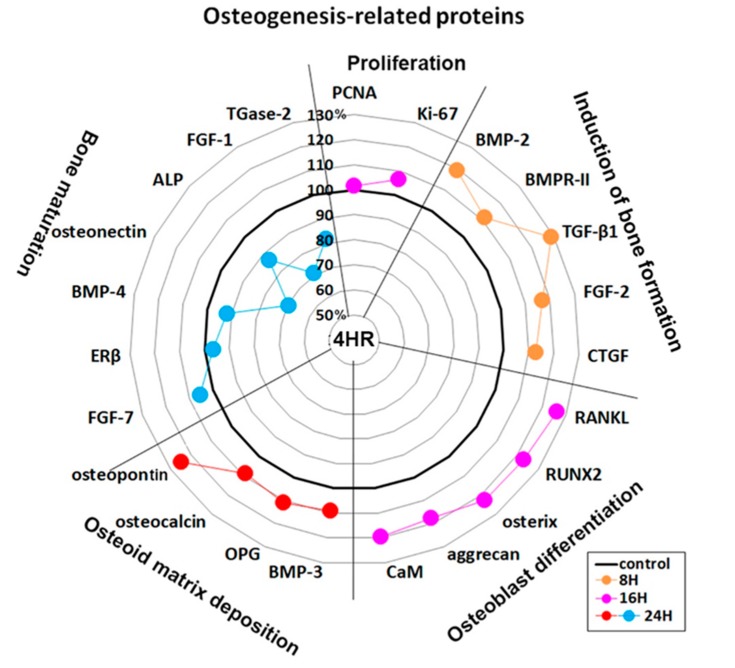
Star plot of global protein expression in Saos-2 cells treated with 4HR for 24 h. The expression level (%) of osteogenesis-related proteins (*n* = 23) were sequentially dominant (in accordance with the four stages of osteogenesis) at 8 (orange dots), 16 (pink dots), and 24 h (red and blue dots) after 4HR treatment compared to the non-treated control.

An examination of changes in global protein expression in osteogenesis-related proteins (*n* = 23) showed a sequential pattern of dominance during 24 h of 4HR treatment versus the non-treated control (Figure 4). The proteins relevant to the induction of bone formation (BMP-2, BMPR-II, TGF-β1, FGF-2, and CTGF) were upregulated at 8 h, and the proteins relevant to osteoblast differentiation (RANKL, RUNX2, osterix, aggrecan, and CaM) were upregulated at 16 h. While the proteins relevant to osteoid matrix deposition (BMP-3, OPG, osteocalcin, and osteopontin) were upregulated at 24 h, the proteins relevant to bone maturation (ERβ, BMP-4, osteonectin, ALP, FGF-1, and TGase-2) were still downregulated at 24 h. These data suggested that 4HR efficiently induced bone formation in Saos-2 cells by sequential overexpression specific to the stages of osteogenesis.

### 2.3. Application of 4HR Accelerates Tooth Movement

Control group received solvent only. Group A received low dosage of 4HR (1.28 mg/kg) and Group B high dosage of 4HR (128 mg/kg). The distance of tooth movement at day 7 was 0.24 ± 0.84 mm, 0.92 ± 1.00 mm, and 0.89 ± 0.61 mm in the control, Group A, and Group B, respectively (Table 1). The differences between the groups were not statistically significant. At day 14, the distance of tooth movement was 1.98 ± 1.12 mm, 2.63 ± 0.68 mm, and 2.90 ± 0.42 mm in the control, Group A, and Group B, respectively. There was a significant difference among the groups (*p* = 0.043), with the post hoc test showing the difference between the control group and Group B to be statistically significant (*p* = 0.046). These results were in accord to those in the radiogram (Supplementary Figure S1).

**Figure 5:**
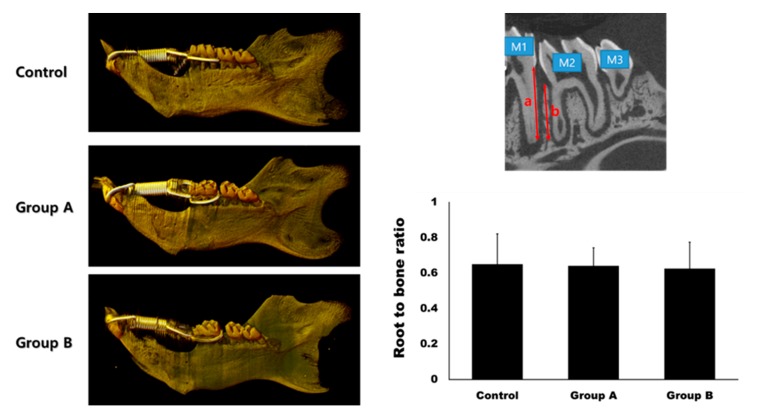
Micro-computerized tomogram. The gap between the first molar (M1) and the second molar (M2) was much wider in Group A and B compared to the control group at day 14. The root-to-bone ratio was measured at the distal surface of the distal root of M1. It was calculated as the ratio between the root length (**a**) and bone height (**b**). There was no significant difference in the root-to-bone ratio among the groups (*p* > 0.05).

The root-to-bone ratio (ratio of the distal root of the first molar to the interdental bone) was 0.65 ± 0.17, 0.64 ± 0.10, and 0.63 ± 0.15 in the control, Group A, and Group B, respectively (Figure 5). The differences between the groups were not statistically significant (*p* > 0.05).

**Figure 6:**
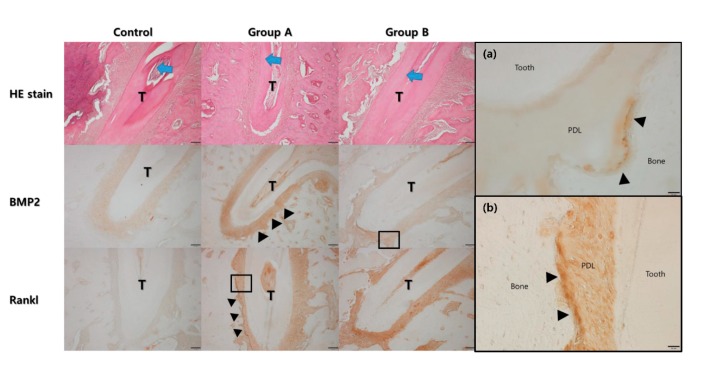
Histological analysis under the hematoxylin and eosin (HE) stain. The direction of tooth movement is indicated by the blue arrow at the central root of the first molar (T). The width of the periodontal ligament space was narrower on the compression side than on the tension side. The expression level of bone morphogenic protein-2 was higher in Group A and B compared to the control group. The expression level was higher on the tension side (arrow heads). The expression level of RANKL was also higher in Group A and B compared to the control group. The expression level was higher on the compression side (arrow heads). (**a**) High magnification views for BMP2 of Group B showed the expression was mainly found in the cells lined on the tension side of the alveolar bone (arrow heads, original magnification ×400) (PDL: periodontal ligament). (**b**) High magnification views for RANKL of Group A showed the expression was mainly found in the cells lined on the compression side of the alveolar bone (arrow heads, original magnification ×400).

In the hematoxylin and eosin stain (HE), the width of the periodontal ligament was narrower on the compression side than on the tension side (Figure 6). The expression of BMP-2 and RANKL were higher in Groups A and B compared to the control group. The expression of BMP-2 was higher on the tension side than on the compression side in Group A, while the expression of RANKL was higher on the compression side than on the tension side in Group A. A similar trend was observed in Group B. However, the expression level of RANKL was much higher than in Group A.

**Figure 7:**
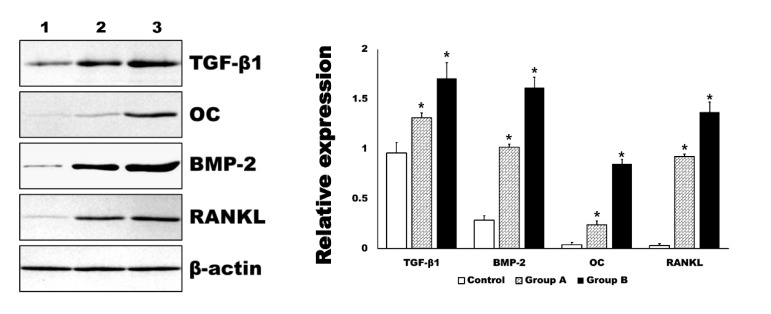
Western blot for tissue samples. The level of transforming growth factor-β1 (TGF-β1), osteocalcin (OC), bone morphogenic protein-2 (BMP-2), and receptor activator of nuclear factor kappa-Β ligand (RANKL) were significantly increased in 4HR administered groups (Group A and B, * *p* < 0.001). The increasing of each marker was dependent on the applied dosage of 4HR (1: control for solvent only, 2: Group A-1.28 mg/kg 4HR administered, 3: Group B-128 mg/kg 4HR administered).

In the Western blot analysis, the expression level of BMP-2 and RANKL were in accord to those of immunohistochemistry (Figure 7). In addition, the expression level of TGF-β1 and OC were also significantly increased by 4HR administration compared to the control group (*p* < 0.001). The relative expression level to β-actin for each protein is shown in the graph (Figure 7).

### 2.4. Plasma Level of Bone Turnover Markers

**Figure 8:**
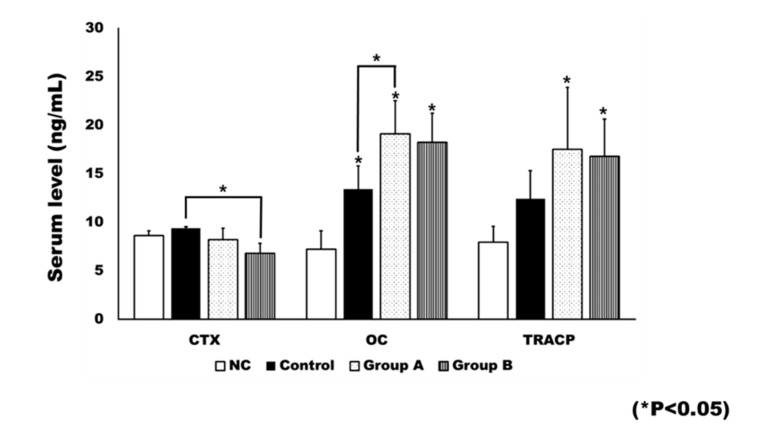
The plasma level of bone turnover markers. The level of c-terminal cross linking telopeptide (CTX) was generally decreased after 4HR treatment. The CTX level in Group B was significantly lower than that of the control (* *p* < 0.05). The level of osteocalcin (OC) was significantly increased after ovariectomy (* *p* < 0.05). In addition, 4HR administration further increased the OC level. When compared to the control group, Group A showed a significantly higher OC level (* *p* < 0.05). The tartrate-resistant acid phosphatase (TRACP) level was also increased after ovariectomy. When compared to the untreated control, both Groups A and B showed significantly increased TRACP levels (* *p* < 0.05).

Rats in the negative control (NC) group did not receive an ovariectomy. Ovariectomy was associated with significant changes in bone turnover markers (Figure 8). The OC level was significantly different between groups (*p* < 0.001). When compared to the NC group, the control group (*p* = 0.004), Group A (*p* <0.001), and Group B (*p* = 0.001) showed significantly higher levels of OC. Group A had a significantly higher level of OC than the control group (*p* = 0.029).

Significant differences were observed between groups in terms of bone resorption markers, including c-terminal cross linking telopeptide (CTX; *p* = 0.010) and TRACP-5p (*p* = 0.002). The post hoc test showed the CTX level of Group B to be significantly lower than that of the control group (*p* = 0.007). The TRACP-5p level was significantly higher in both Group A (*p* = 0.003) and Group B (*p* = 0.009), compared to the NC group.

## 3. Discussion

In this study, 4HR application increased osteogenic markers such as TGF-β1, BMP-2, BMP-4, AP, OC, OP, type I collagen, and RUNX2 in Saos-2 cells (Figure 1, Figure 2 and Figure 4). These findings were confirmed in both Western blot and immunoprecipitation high-performance liquid chromatography (IP-HPLC) analyses (Figure 1, Figure 2 and Figure 4). The administration of 4HR to ovariectomized rats resulted in significantly greater degrees of tooth movement than in the untreated control (Table 1, *p* < 0.05). The administration of 4HR increased the blood levels of both OC and TRACP (Figure 8). In spite of a greater amount of tooth movement, the bone level on the tension side was similar to that in the untreated control (Figure 5). 4HR administration was found to be beneficial for orthodontic tooth movement by accelerating both bone formation and resorption.

Ovariectomy results in an estrogen deficiency, which is associated with increased osteoclast cell differentiation and bone turnover [21,22]. The activation of osteoclasts is a basic requirement for accelerated orthodontic tooth movement [8,9]. Orthodontic tooth movement is significantly increased by ovariectomy [24,25]. All groups exhibited tooth movement after the application of the orthodontic appliance. The TRACP level was higher in all groups receiving an ovariectomy, compared to the NC group. If the rate of bone formation is not equivalent to that of bone resorption, complications such as periodontal lesions or root resorption may occur [26,27]. Thus, both bone resorption and bone formation are important for complication-free orthodontic tooth movement [26].

In this study, the blood CTX level was not significantly increased by ovariectomy (Figure 8), and only the control group showed a higher average CTX value than the NC group (Figure 8). The blood CTX level is an indirect indicator of osteoclast activity [28]. It is increased when the balance between bone formation and resorption is tipped towards the latter [28]. As 4HR administration increased bone formation markers such as OC and BMP-2 (Figure 1, Figure 2, Figure 7 and Figure 8), the balance may have been tipped towards bone formation. The dosage-dependent change of serum markers was not prominent like tissue samples after 4HR administration (Figure 7 and Figure 8). Serum reflects systemic changes and does not confine to bone activity. Thus, they should be considered as Supplementary Data. As a consequence, the ratio between root length and bone height was similar to the control group, in spite of accelerated tooth movement (Figure 5).

The osteogenesis-related proteins were expressed in a stage-specific manner in 4HR-treated Saos-2 cells, in accordance with the sequence of bone formation induction, osteoblast differentiation, osteoid matrix deposition, and bone maturation (Figure 3 and Figure 4). At 8 h after 4HR administration, overexpression of BMP-2, BMPR-II, TGF-β1, FGF-2, and CTGF was induced, which synergistically contributed to bone formation. Thereafter, the expression levels of these proteins decreased to the level of the non-treated control at 16 and 24 h (Figure 4). At 16 h, 4HR induced overexpression of RANKL (osteoclast differentiation factor) [29], RUNX2 (a key transcription factor associated with osteoblast differentiation) [30], osterix (a transcription factor for osteoblast differentiation) [31], aggrecan (a cartilage-specific proteoglycan core protein) [32], and calmodulin-dependent kinase II (a key regulator of osteoblast differentiation) [33], which co-operatively stimulated osteoblast differentiation. At 24 h, 4HR induced overexpression of BMP-3 (a negative regulator for bone density) [34], OPG (a regulator for bone density) [35], osteocalcin (a calcium-binding protein) [36], and osteopontin (bone sialoprotein I) [37], which simultaneously regulated osteoid matrix deposition. These results corresponded with those of the Western blot (Figure 1 and Figure 2).

In the histological analysis, increased expression of BMP-2 and RANKL were evident in Groups A and B (Figure 6). Notably, the expression of BMP-2 was more attenuated on the tension side compared to the compression side (Figure 6). BMP-2 is associated with bone regeneration and is a member of the TGF-β family [38]. In cellular experiments, 4HR has been demonstrated to increase the TGF-β1 expression level [19]. As bone deposition is required on the tension side during orthodontic tooth movement, an increased expression of BMP-2 at this site may be attributed to its role in active bone formation. RANKL is a marker for osteoclasts [29]. The expression of RANKL was mainly observed on the compression side, as opposed to the tension side (Figure 6), indicating its role in active bone resorption. These findings corresponded to the results of the Western blot for tissue samples and blood sample analysis (Figure 7 and Figure 8).

Some limitations are acknowledged in this study. First, the rats were too young to show estrogen deficiency after ovariectomy. Compensatory estrogen secretion from the adrenal glands seemed to be much higher in all groups receiving an ovariectomy, compared to the NC group (Supplementary Figure S2) [39]. In spite of this limitation, both bone formation and resorption markers were elevated following the ovariectomy (Figure 8). In addition, serum OC levels were also higher in the ovariectomized rats compared to the NC group [40] (Figure 8). Thus, the original objective of the surgical procedure (i.e., to increase bone turnover rate) was achieved. Second, the mechanism of osteoclast activation by 4HR administration was unclear. Increased levels of TRACP and RANKL, as well as rapid tooth movement, demonstrate the activation of osteoclasts, regardless of the surgical effects. As TGF-β1 is a master cytokine for bone metabolism, an increased expression of TGF-β1 may contribute to osteoclast activation. Furthermore, TRACP activity in pre-osteoclastic RAW264.7 cells is increased in the presence of TGF-β1 [41], and the expression of TGF-β1 is increased in RAW264.7 cells after 4HR administration [19]. However, this requires further clarification in a future study. Third, the difference in dosage of 4HR between Groups A and B was greater than 100-fold. Additional studies are required to determine an optimal dosage within this range. Fourth, the orthodontic force generated by closed coil spring is progressively reduced as its deactivation. Accordingly, some animals showed fully deactivated spring and further movement was impossible. This attenuated the difference according to the applied dosage. Rat model has been widely used for the orthodontic movement because of similar characteristics with humans [42]. However, any animal study has inherent limitations [43]. Therefore, clinical application of 4HR for the orthodontic treatment should be careful.

## 4. Materials and Methods

### 4.1. Cellular Experiment and Western Blot

Saos-2 cells (Korean Cell Line Bank No. 30085, Seoul, Korea) were suspended in RPMI 1640 supplemented with 10% fetal bovine serum (Euroclone, Milano, Italy), 50 U/mL of penicillin G, 50 μg/mL of streptomycin sulfate, 2 g/L sodium carbonate, and 0.11 g/L sodium pyruvate. 4HR was purchased from Sigma-Aldrich (St. Louis, MO, USA).

To analyze the protein expression level of TGF-β1, BMP-2, BMP-4, AP, OC, OP, type I collagen, and RUNX2 (Santa Cruz Biotechnology, Santa Cruz, CA, USA), Saos-2 cells were treated with 1, 10, and 100 μM of 4HR. The control consisted of Saos-2 cells treated by solvent only. Proteins were collected 2, 8, and 24 h after treatment. Collected proteins were mixed with a sodium dodecyl sulfate buffer and denatured by heating. Denatured proteins were electrophoresed on 10% polyacrylamide gels. The gels were transferred to polyvinylidene difluoride membranes. After blocking, the membranes were probed with primary antibodies (dilution ratio = 1:500). Blots were imaged and quantified using a ChemiDoc XRS system (Bio-Rad Laboratories, Hercules, CA, USA).

### 4.2. IP-HPLC

The basic principle of IP-HPLC is similar to the enzyme-linked immunosorbent assay (ELISA). However, IP-HPLC uses protein A/G agarose beads in buffer solution and UV spectroscopy to determine protein concentrations. In this study, 100 μg of protein from a Saos-2 cell culture were subjected to immunoprecipitation. Protein A/G agarose columns (Amicogen, Jinju, Korea) were separately pre-incubated with 1 μg of 96 different antisera (Supplementary Table S1). The subsequent procedure has been detailed in our previous publications [19,44,45] and the Supplementary File.

### 4.3. Animals and Experimental Design

Six-week-old Crl:CD (Sprague-Dawley) specific pathogen-free (SPF)/viral antibody-free (VAF) outbred female rats (Orientbio Inc., Sungnam, Korea) were used in this study. All procedures were performed in accordance with guidelines for laboratory animal care and were approved at July 15, 2019 by the Gangneung-Wonju National University for animal research (GWNU-2019-17). Thirty rats (2–3 rats per cage) were housed and fed as previously described [14,18]. All rats were acclimatized for 1 week prior to experimentation. Each rat was ovariectomized on both sides and rested for 2 weeks after the operation. Blood samples were obtained to monitor changes in estrogen levels.

**Figure 9:**
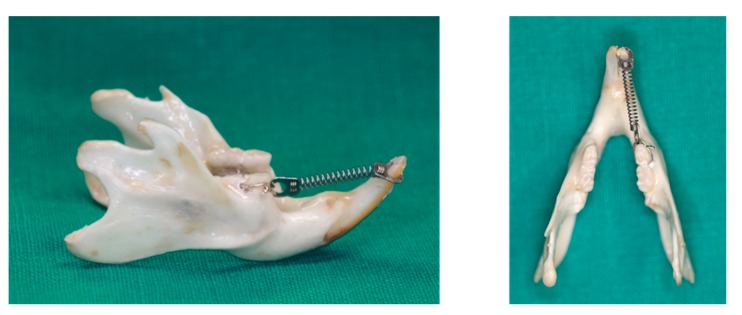
Application of an orthodontic appliance in a rat model.

All rats received an orthodontic appliance consisting of an 8-mm nickel-titanium closed coil spring (Jinsung, Seoul, Korea) stretched between the right mandibular first molars and right mandibular incisors (Figure 9). The ring (connected to the spring) was inserted onto the incisor and fixed with resin. The other side of the spring was ligated to the first molar, with holes drilled lateral to the distal side of the molar at the gingival level, using a round bur. The spring provided a force of 120 g, as measured with the use of a force gauge (Tokyo-Seiki, Tokyo, Japan) The appliance was left in place without reactivation for 2 weeks to induce mesial movement of the mandibular first molar.

The rats were divided into 3 groups with 10 rats each. There were 2 experimental groups (low and high dosage group). The rats in the low dosage group received 1.28 mg/kg 4HR via subcutaneous injection on day 0, 7, and 14 (Group A). The rats in the high dosage group received 128 mg/kg 4HR via subcutaneous injection on day 0, 7, and 14 (Group B). The rats in the control group received a solvent only. The distance between the mesial surface of the first molar and the distal surface of the incisor was measured on day 0, 7, and 14 using wire under inhalation anesthesia. The distance between points marked on wire was measured with Vernier caliper. The amount of tooth movement was defined as the change in distance at each observation compared to day 0. Additionally, the final gap between the first and the second molars was also measured using a radiogram and software (SigmaScan Pro 5, SPSS Inc., Chicago, IL, USA).

Two hours after the final injection on day 14, blood was sampled and all rats were then sacrificed. For comparative purposes, sham operative animals (*n* = 5) were included in the blood sample analysis, and served as the negative control. Blood OC (CAT#: E-EL-R1456), CTX (CAT#: E-EL-R0243), and TRACP-5b (CAT#: E-EL-R0628) measuring kits were purchased from Elabscience (Houston, TX, USA). For the ELISA or chemiluminescent immunoassay (CLIA) analysis, whole blood samples were centrifuged and the plasma collected. The plasma was mixed with protein lysis buffer at a 1:1 volume ratio. These mixtures were used for ELISA analysis. The subsequent procedures were conducted in accordance with the manufacturer’s protocol. A biopsy was obtained from the mandible, and micro-computerized tomography and histological analyses were performed.

### 4.4. Micro-Computerized Tomography and Histological Analysis

The hemi-mandibles were sent to Genoss (Seoul, Korea) for analysis with micro-computerized tomography. The subsequent procedures were conducted in accordance with our previous publications. In brief, prepared samples were loaded on SkyScan1173 (Bruker, Kontich, Belgium). The source voltage was 130 kV and the image pixel size was 13.85 μm. In the cross-cut image, the root-to-bone ratio was measured at the distal surface of the distal root of the first molar. Three-dimensional reconstruction was done with the software provided by the manufacturer.

Following micro-computerized tomography, the samples underwent decalcification with decalcifying solution-lite (Sigma Aldrich, St. Louis, MO, USA). After decalcification, samples were embedded in paraffin and sectioned at a thickness of 10 μm. The specimens were stained with HE. The antibodies for immunostaining were the same as those used in the Western blot and IP-HPLC. After deparaffinization, trypsin treatment was performed for antigen retrieval, and an endogenous peroxidase block was carried out. After washing, a protein block was applied for 1 h. Antibodies for BMP-2 or RANKL were then incubated in humidified chambers at 4 °C overnight. After washing, Envision (Dako, Glostrup, Denmark) was applied, and diaminobenzidine was used for colorization. Photos of the slides were taken without counterstaining.

### 4.5. Western Blot for Tissue Samples

The opposite mandibles, that were not used for tooth movement, were placed into micro-test tubes, and stored in the deep freezer (*n* = 10 for each group). The tissues were vigorously homogenized in a tissue protein extraction reagent buffer with a protease inhibitor cocktail, and Western blot analysis was performed as described above. The expression level of each protein was measured using image analysis program. The expression level of β-actin was set as 1. The relative expression level of β-actin was calculated and compared.

### 4.6. Statistical Analysis

Proportional data (%) of the experimental and control groups were plotted into line graphs and star plots, and analyses were repeated 2 to 6 times until the standard deviations were < ±5%. Line graphs revealed the similarities in expression patterns between the relevant proteins, and star plots revealed the differences in expression levels of the whole objective proteins. Results were analyzed using the chi-squared test. The expression of control housekeeping proteins (i.e., β-actin, α-tubulin, and GAPDH) were nonresponsive (≤5%) to 12, 24, and 48 h of 4HR treatment.

The relative protein expression level in the western blot among groups was compared with ANOVA. Using the post hoc test, the difference between groups was analyzed. The significance level was set as 0.05.

## 5. Conclusions

In this study, the administration of 4HR resulted in an increased expression of osteogenic markers in Saos-2 cells. The administration of 4HR in ovariectomized rats also resulted in accelerated orthodontic tooth movement and increases in the levels of both bone formation markers (OC and BMP-2) and bone resorption markers (TRACP and RANKL).

## Figures and Tables

**Table 1:** Intergroup comparison of distance of tooth movement.

Group	Day 7	Day 14
Control group	0.24 ± 0.84 mm	1.98 ± 1.12 mm
Experimental Group A	0.92 ± 1.00 mm	2.63 ± 0.68 mm
Experimental Group B	0.89 ± 0.61 mm	2.90 ± 0.42 mm *

* *p* < 0.05, comparison between the experimental groups and control group at each time point. The values are presented as mean ± SD.

## Supplementary Materials

SupplementaryMaterials can be found at https://www.mdpi.com/1422-0067/21/4/1526/s1.

## Abbreviations

AP	Alkaline phosphatase
BMP	Bone morphogenic protein
BMPR-II	Bone morphogenetic protein receptor-II
CaM	Calmodulin
CTGF	Connective tissue growth factor
ELISA	Enzyme-linked immunosorbent assay
ERβ	Estrogen receptor β
FGF-2	Fibroblast growth factor-2
4HR	4-Hexylresorcinol
IP-HPLC	Immunoprecipitation high-performance liquid chromatography
OC	Osteocalcin
OP	Osteopontin
Runx2	Runt-related transcription factor 2
TRACP	Tartrate-resistant acid phosphatase
TGF-β1	Transforming growth factor-β1
TGase-2	Transglutaminase-2
