# Supplementary material for: The Administration of 4-Hexylresorcinol Accelerates Orthodontic Tooth Movement and Increases the Expression Level of Bone Turnover Markers in Ovariectomized Rats
Source: Int J Mol Sci. 2020 Feb 24;21(4):1526. doi: 10.3390/ijms21041526 (PMC7073238; doi:10.3390/ijms21041526)

The Administration of 4-Hexylresorcinol Accelerates Orthodontic Tooth Movement and Increases the Expression Level of Bone Turnover Markers in Ovariectomized Rats

Kwang-Hyo Choi[1], Dae-Won Kim[2], Suk Keun Lee[3], Seong-Gon Kim[4,*], and Tae-Woo Kim[1,*]

[1]Dept. of Orthodontics, College of Dentistry, Seoul National University, Seoul 3080, Republic of Korea; joshuakh@hotmail.com (C.K.H.)

[2]Dept. of Oral Biochemistry, College of Dentistry, Gangneung-Wonju National University, Gangneung 28644, Republic of Korea; kimdw@gwnu.ac.kr

[3]Department of Oral Pathology, College of Dentistry, Gangneung-Wonju National University, and Institute of Oral Science, Gangneung 28644, Republic of Korea; sukkeunlee@hanmail.net

[4]Dept. of Oral and Maxillofacial Surgery, College of Dentistry, Gangneung-Wonju National University, Gangneung 28644, Republic of Korea

*Correspondence: kimsg@gwnu.ac.kr; Tel.:+82-33-640-2468 (K.S.G.) and taewoo@snu.ac.kr; Tel:+82-2-2072-2678 (K.T.W.)

Supplementary Table 1. Antibodies used in the study.

Protein	No.	Antibodies
Growth factor-related protein	10	FGF-1[*], FGF-2[*], CTGF, TGF-β1[#], TGF-β2[*], TGF-β3[*], SMAD4[*], SMAD2/3, p-SMAD4, PDGF-A[*],
RAS signaling proteins	22	NRAS[$], KRAS[$], HRAS, PI3K, pAKT1/2/3, RAF-B[*], JNK-1[*], p-JNK-1, ERK-1[*], p-ERK-1[$], Rab 1[*], STAT3, p38[*], p-p38[*]
NFkB signaling proteins	12 (2)	NFkB[*], IKK[*], GADD45[*], GADD153[*], mTOR[@], NRF-2[*], PGC-1α, SRC-1[*], MDR, AMPK (p38[*], p-p38[*])
Inflammatory proteins	20	IL-10[*], lysozyme[*], granzyme, lactoferrin, M-CSF, Pdcd-1/1, HCAM, ICAM-1, COX2[*], versican, TNFα[@], IL-6[*], LTA4H[&], CXCR4, cathepsin C, cathepsin G[*], MCP-1, CD68, CD99, TLR3
Apoptosis-related proteins	20	p53[*], BAD[*], BAK[*], BAX, BCL2, AIF[*], APAF-1, caspase 9[*], c-caspase 9[*], PARP-1[*], c-PARP-1[*], FASL[*], FAS[*], FADD[*], FLIP[*], BID, c-caspase 8[*], c-caspase-10, caspase 3[*], c-caspase 3[*]
Angiogenesis-related proteins	14 (3)	HIF-1α[&], angiogenin[$], VEGF-A[*], VEGF-C[*], vWF[$], CMG2[$], FLT-4[$], LYVE-1[*], MMP-2, MMP-10, PECAM-1 (FGF-2, PDGF-A, ICAM-1)
Control housekeeping proteins	3	α-tubulin[*], β-actin[*], GAPDH[*]
Total	101 (5)	

[*] Santa Cruz Biotechnology, CA, USA; [#] DAKO, Denmark; [$] Neomarkers, CA, USA; [@] ZYMED, CA, USA; [&]Abcam, Cambridge, UK; the number of antibodies overlapped; ( ).

Abbreviations: AIF; apoptosis inducing factor, AMPK; AMP-activated protein kinase, pAKT; v-akt murine thymoma viral oncogene homolog, p-Akt1/2/3 phosphorylated (p-Akt, Thr 308), BAD; BCL2 associated death promoter, BAK; BCL2 antagonist/killer, BAX; BCL2 associated X, CMG2: capillary morphogenesis protein 2, COX-2; cyclooxygenase-2, CTGF connective tissue growth factor, CXCR4; C-X-C chemokine receptor type 4, FADD; FAS associated via death domain, FAS; CD95/Apo1, FASL; FAS ligand, FGF-1; fibroblast growth factor-1, FLIP; FLICE-like inhibitory protein, FLT-4; Fms-related tyrosine kinase 4, GADD45; growth arrest and DNA-damage-inducible 45, GAPDH; glyceraldehyde 3-phosphate dehydrogenase, HCAM (CD44); homing cell adhesion molecule, HDAC-10; histone deacetylase 10, HIF-1α: hypoxia inducible factor-1α, HRAS; GTPase HRas, HSP-70; heat shock protein-70, ICAM (CD54); intercellular adhesion molecule 1, IKK; ikappaB kinase, IL-1; interleukin-1, JNK-1; Jun N-terminal protein kinase, KRAS; V-Ki-ras2 Kirsten rat sarcoma viral oncogene homolog, LTA4H; leukotriene A4 hydrolase, LYVE-1: lymphatic vessel endothelial hyaluronan receptor 1, MCP-1; monocyte chemotactic protein 1, M-CSF; macrophage colony-stimulating factor, MDR; multiple drug resistance, MMP-2; matrix metalloprotease-2, mTOR; mammalian target of rapamycin, NCAM (CD56); neural cell adhesion molecule 1, NF-1; neurofibromin 1, NFkB; nuclear factor kappa-light-chain-enhancer of activated B cells, NRAS; neuroblastoma RAS Viral Oncogene homolog, NRF2; nuclear factor (erythroid-derived)-like 2, PARP-1; poly-ADP ribose polymerase 1, c-PARP-1; cleaved-PARP-1, Pdcd-1/1 (CD279); programmed cell death protein 1, PDGF-A: platelet-derived growth factor-A, PECAM-1 (CD31); platelet endothelial cell adhesion molecule-1, PGC-1α; peroxisome proliferator-activated receptor gamma coactivator 1-α, PI3K; phosphatidylinositol-3-kinase, PTEN; phosphatase and tensin homolog, Rab 1; Rab GTPases, RAF-B; v-Raf murine sarcoma viral oncogene homolog B, SMAD4; mothers against decapentaplegic, drosophila homolog 4, SRC1; steroid receptor coactivator-1, STAT3; signal transducer and activator of transcription-3, TGF-β1; transforming growth factor-β1, TNFα; tumor necrosis factor-α, VEGF-A vascular endothelial growth factor A, vWF: von Willebrand factor.

Detailed Procedures for IP-HPLC

Protein samples were mixed with 5 mL of binding buffer (150mM NaCl, 10mM Tris pH 7.4, 1mM EDTA, 1mM EGTA, 0.2mM sodium vanadate, 0.2mM PMSF, and 0.5% NP-40) and incubated in protein A/G agarose (Amicogen, Korea) columns on a rotating stirrer for 1 hour at 4°C. After washing columns with PBS (phosphate-buffered saline solution), target proteins were eluted using 150µL of IgG elution buffer (Pierce, USA). Immunoprecipitated proteins were analyzed using an HPLC unit (1100 series, Agilent, USA) equipped with a reverse phase column and a micro-analytical detector system (SG Highteco, Korea). Elution was performed using 0.15M NaCl/20% acetonitrile solution at 0.4 mL/min for 30 min, and proteins were detected using an unltraviolet spectrometer at 280 nm. Control and experimental samples were run sequentially to allow comparisons. For IP-HPLC, whole protein peak areas (mAU*s) were calculated after subtracting negative control antibody peak areas, and square roots of protein peak areas were calculated to normalize concentrations. Protein percentages in total proteins in experimental and control groups were plotted. Results were analyzed using the chi-squared test.

The housekeeping proteins β-actin, α-tubulin, and glyceraldehyde 3-phosphate dehydrogenase (GAPDH) were used as internal controls. Expressional changes of housekeeping proteins were adjusted to < ±5% using a proportional basal line algorithm. Protein expressional changes of ≤ ±5%, ±5-10%, ±10-20%, and ≥±20% change were defined as minimal, slight, meaningful, or marked, respectively.

Supplementary Figure 1. The gap between the first and the second molar measured in radiogram

The gap between M1 and M2 at day 14 was 0.22 ± 0.19 mm, 0.44 ± 0.23 mm, and 0.51 ± 0.21 mm in the control, Group A, and Group B, respectively. There was a significant difference among the groups (P = 0.011). In post hoc test, the difference between the control group and Group B to be statistically significant (P = 0.012).

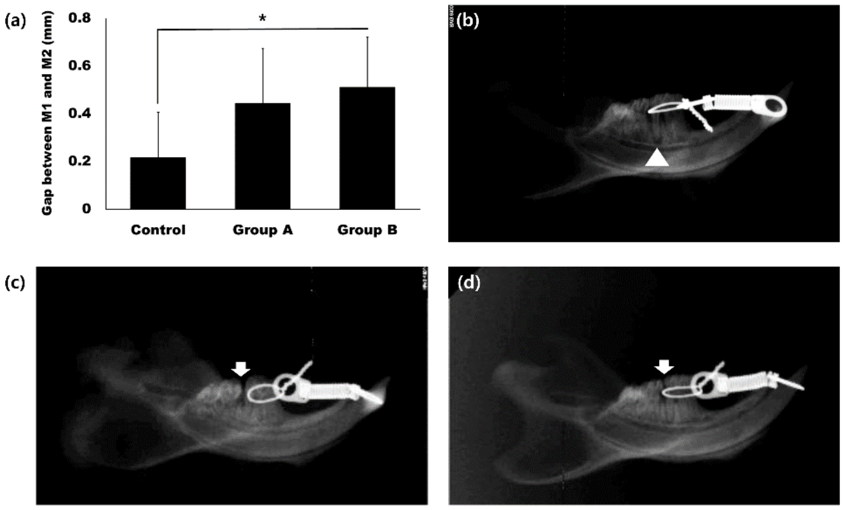

(*P<0.05 compared to control group)

Supplementary Figure 2. Blood estradiol level

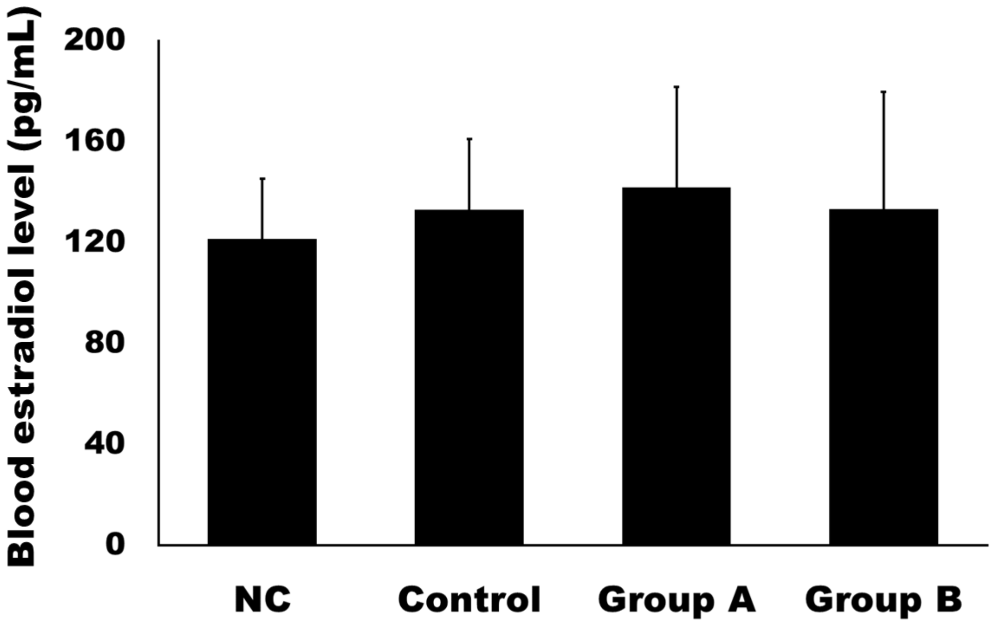

The average value of blood estradiol was slightly increased after ovariectomy. However, the difference among groups was not statistically significant ($P>0.05$).

Supplementary Figure 3. High magnification view of Figure 6

4HR induced BMP-2 expression (arrow heads) in the tension side of Group B tissue samples (original magnification x400)

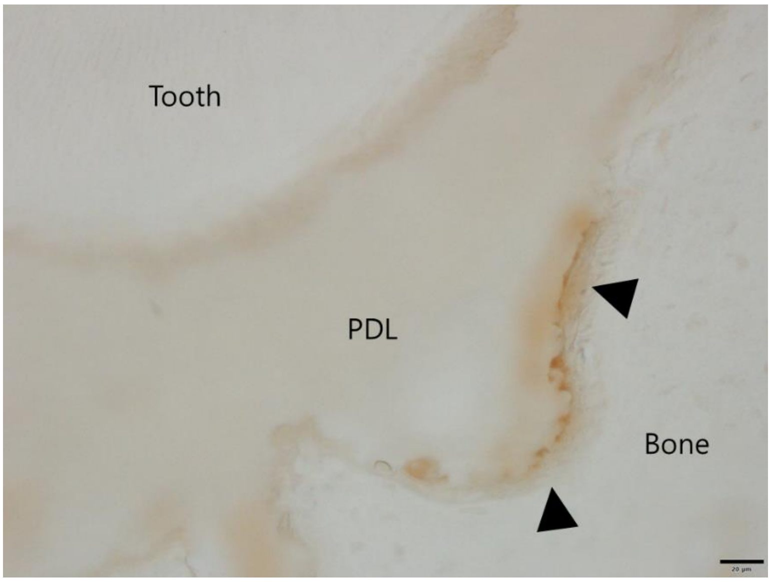

(PDL: periodontal ligament)

4HR induced RANKL expression (arrow heads) in the compression side of Group A tissue samples (original magnification x400)

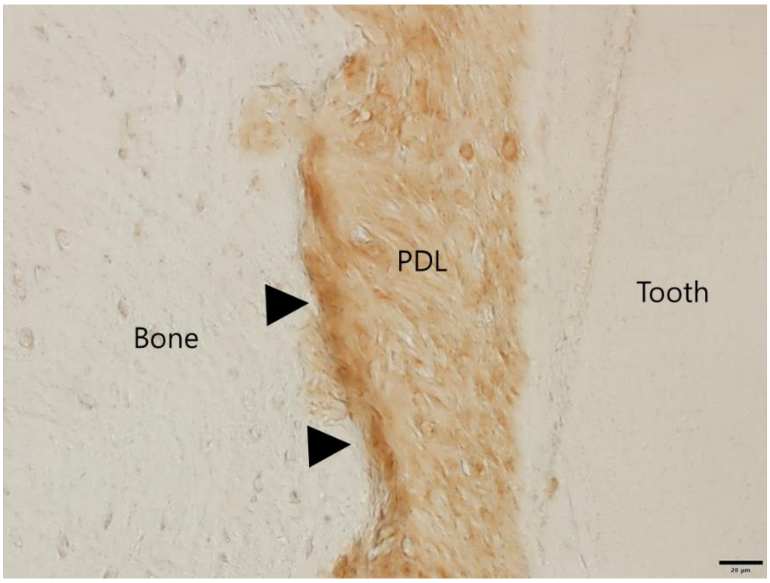

(PDL: periodontal ligament)

Supplementary Figure 4. Full length blot of Figure 1

4HR induced TGF-β1, BMP-2, and BMP-4 expression

A. β-actin (from left lane to right, **1:** no-treatment, **2:** 1 μM 4HR at 2 h, **3:** 10 μM 4HR at 2 h, **4:** 100 μM 4HR at 2 h, **5:** 1 μM 4HR at 8 h, **6:** 10 μM 4HR at 8 h, **7:** 100 μM 4HR at 8 h, **8:** 1 μM 4HR at 24 h, **9:** 10 μM 4HR at 24 h, **10:** 100 μM 4HR at 24 h)

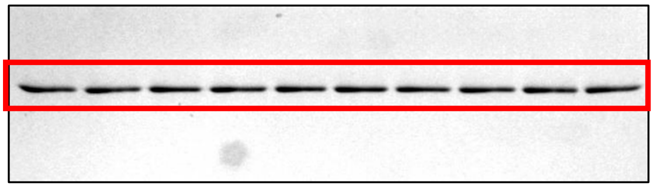

B. TGF-β1 (from left lane to right, **1:** no-treatment, **2:** 1 μM 4HR at 2 h, **3:** 10 μM 4HR at 2 h, **4:** 100 μM 4HR at 2 h, **5:** 1 μM 4HR at 8 h, **6:** 10 μM 4HR at 8 h, **7:** 100 μM 4HR at 8 h, **8:** 1 μM 4HR at 24 h, **9:** 10 μM 4HR at 24 h, **10:** 100 μM 4HR at 24 h)

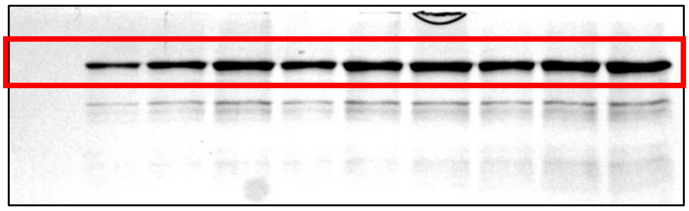

C. BMP-2 (from left lane to right, **1:** no-treatment, **2:** 1 μM 4HR at 2 h, **3:** 10 μM 4HR at 2 h, **4:** 100 μM 4HR at 2 h, **5:** 1 μM 4HR at 8 h, **6:** 10 μM 4HR at 8 h, **7:** 100 μM 4HR at 8 h, **8:** 1 μM 4HR at 24 h, **9:** 10 μM 4HR at 24 h, **10:** 100 μM 4HR at 24 h)

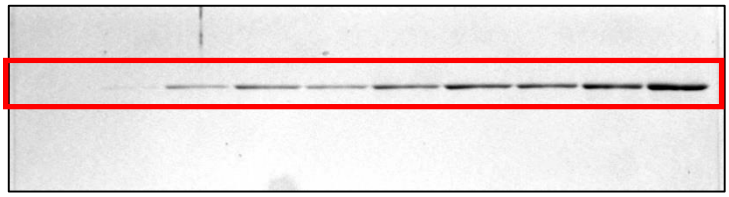

D. BMP-4 (from left lane to right, **1:** no-treatment, **2:** 1 μM 4HR at 2 h, **3:** 10 μM 4HR at 2 h, **4:** 100 μM 4HR at 2 h, **5:** 1 μM 4HR at 8 h, **6:** 10 μM 4HR at 8 h, **7:** 100 μM 4HR at 8 h, **8:** 1 μM 4HR at 24 h, **9:** 10 μM 4HR at 24 h, **10:** 100 μM 4HR at 24 h)

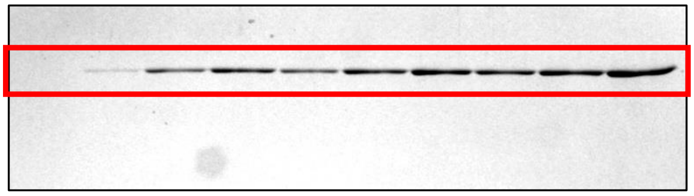

Supplementary Figure 5. Full length blot of Figure 2

4HR induced AP, osteocalcin, osteopontin, type I collagen, and RUNX2 expression

A. β-actin (from left lane to right, **1:** no-treatment, **2:** 1 μM 4HR at 2 h, **3:** 10 μM 4HR at 2 h, **4:** 100 μM 4HR at 2 h, **5:** 1 μM 4HR at 8 h, **6:** 10 μM 4HR at 8 h, **7:** 100 μM 4HR at 8 h, **8:** 1 μM 4HR at 24 h, **9:** 10 μM 4HR at 24 h, **10:** 100 μM 4HR at 24 h)

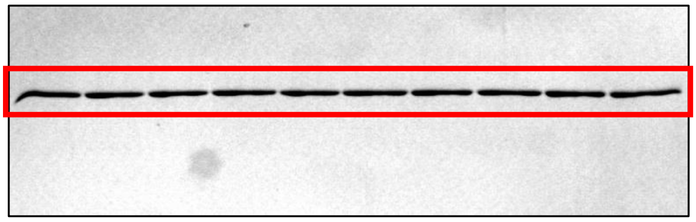

B. AP (from left lane to right, **1:** no-treatment, **2:** 1 μM 4HR at 2 h, **3:** 10 μM 4HR at 2 h, **4:** 100 μM 4HR at 2 h, **5:** 1 μM 4HR at 8 h, **6:** 10 μM 4HR at 8 h, **7:** 100 μM 4HR at 8 h, **8:** 1 μM 4HR at 24 h, **9:** 10 μM 4HR at 24 h, **10:** 100 μM 4HR at 24 h)

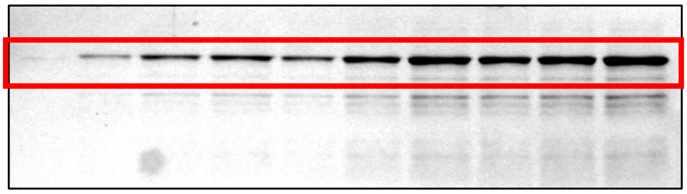

C. Osteocalcin (from left lane to right, **1:** no-treatment, **2:** 1 μM 4HR at 2 h, **3:** 10 μM 4HR at 2 h, **4:** 100 μM 4HR at 2 h, **5:** 1 μM 4HR at 8 h, **6:** 10 μM 4HR at 8 h, **7:** 100 μM 4HR at 8 h, **8:** 1 μM 4HR at 24 h, **9:** 10 μM 4HR at 24 h, **10:** 100 μM 4HR at 24 h)

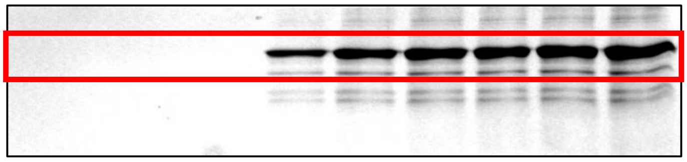

D. Osteopontin (from left lane to right, **1:** no-treatment, **2:** 1 μM 4HR at 2 h, **3:** 10 μM 4HR at 2 h, **4:** 100 μM 4HR at 2 h, **5:** 1 μM 4HR at 8 h, **6:** 10 μM 4HR at 8 h, **7:** 100 μM 4HR at 8 h, **8:** 1 μM 4HR at 24 h, **9:** 10 μM 4HR at 24 h, **10:** 100 μM 4HR at 24 h)

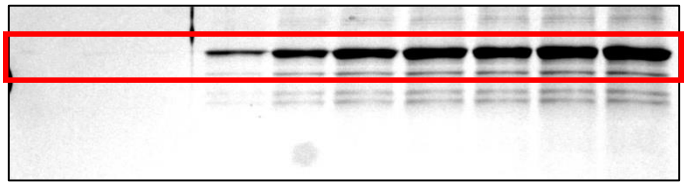

E. Type I collagen (from left lane to right, **1:** no-treatment, **2:** 1 μM 4HR at 2 h, **3:** 10 μM 4HR at 2 h, **4:** 100 μM 4HR at 2 h, **5:** 1 μM 4HR at 8 h, **6:** 10 μM 4HR at 8 h, **7:** 100 μM 4HR at 8 h, **8:** 1 μM 4HR at 24 h, **9:** 10 μM 4HR at 24 h, **10:** 100 μM 4HR at 24 h)

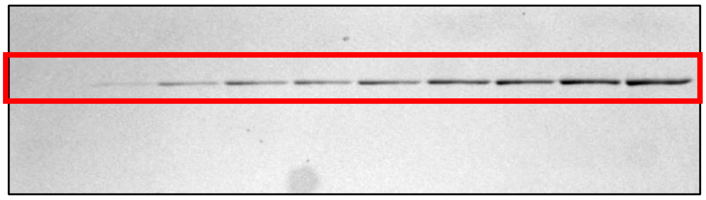

F. RUNX2 (from left lane to right, **1:** no-treatment, **2:** 1 μM 4HR at 2 h, **3:** 10 μM 4HR at 2 h, **4:** 100 μM 4HR at 2 h, **5:** 1 μM 4HR at 8 h, **6:** 10 μM 4HR at 8 h, **7:** 100 μM 4HR at 8 h, **8:** 1 μM 4HR at 24 h, **9:** 10 μM 4HR at 24 h, **10:** 100 μM 4HR at 24 h)

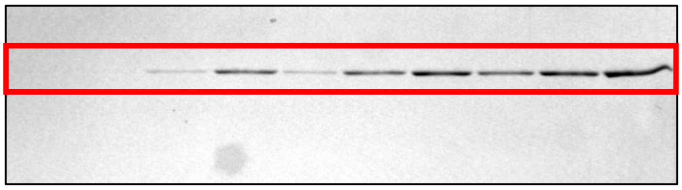

Supplementary Figure 4. Full length blot of Figure 7

4HR induced osteocalcin, BMP-2, TGF-β1, and RANKL expression in the tissue samples

A. β-actin (from left lane to right, **1:** Control – injecting solvent only, **2:** Group A: The rats in the low dosage group received 1.28 mg/kg 4HR via subcutaneous injection. **3:** Group B: The rats in the high dosage group received 128 mg/kg 4HR via subcutaneous injection

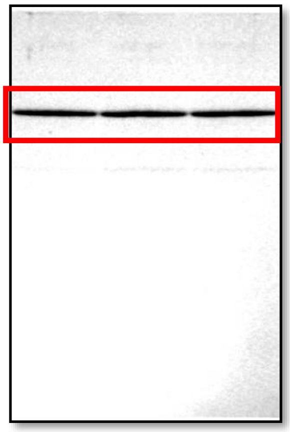

B. Osteocalcin (from left lane to right, **1:** Control – injecting solvent only, **2:** Group A: The rats in the low dosage group received 1.28 mg/kg 4HR via subcutaneous injection. **3:** Group B: The rats in the high dosage group received 128 mg/kg 4HR via subcutaneous injection

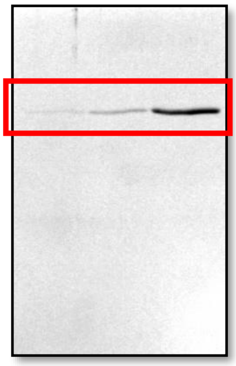

C. TGF-β1 (from left lane to right, **1:** Control – injecting solvent only, **2:** Group A: The rats in the low dosage group received 1.28 mg/kg 4HR via subcutaneous injection. **3:** Group B: The rats in the high dosage group received 128 mg/kg 4HR via subcutaneous injection

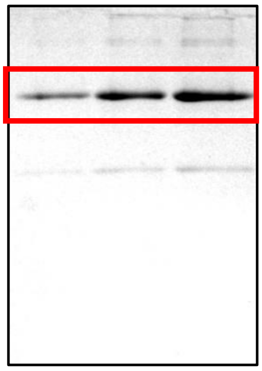

D. BMP-2 (from left lane to right, **1:** Control – injecting solvent only, **2:** Group A: The rats in the low dosage group received 1.28 mg/kg 4HR via subcutaneous injection. **3:** Group B: The rats in the high dosage group received 128 mg/kg 4HR via subcutaneous injection

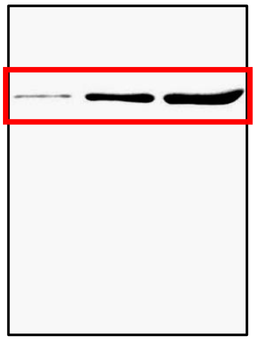

E. RANKL (from left lane to right, **1:** Control – injecting solvent only, **2:** Group A: The rats in the low dosage group received 1.28 mg/kg 4HR via subcutaneous injection. **3:** Group B: The rats in the high dosage group received 128 mg/kg 4HR via subcutaneous injection

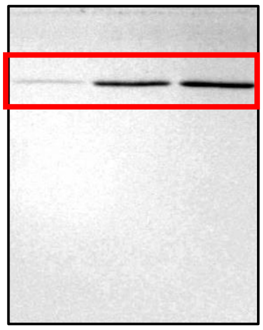

Supplement: Supplementary file 1 [file ijms-21-01526-s001.pdf]
